# Engaging patients to develop a customized digital health companion for periodontitis: Study protocol

**DOI:** 10.3389/froh.2022.1004091

**Published:** 2022-09-12

**Authors:** Lina Weinert, Stefan Listl, Bettina Dannewitz, Oliver Heinze, Tanja Mostovic, Olivier Kalmus, Nihad El Sayed

**Affiliations:** ^1^ Section for Translational Health Economics, Department for Conservative Dentistry, Heidelberg University Hospital, Heidelberg, Germany; ^2^ Chair for Quality and Safety of Oral Health Care, Radboud Institute of Health Sciences, Department of Dentistry, Radboud University Medical Center, Nijmegen, Netherlands; ^3^ Department of Periodontology, Center for Dentistry and Oral Medicine (Carolinum), Johann Wolfgang Goethe-University Frankfurt/Main, Frankfurt/Main, Germany; ^4^ Product Development / Innovation, phellow seven GmbH, Heidelberg, Germany

**Keywords:** digital health, mHealth, mixed-methods, oral health, patient empowerment, patient journey, periodontitis

## Abstract

Periodontitis is a chronic inflammatory disease resulting in the destruction of tooth-supporting tissues. It affects billions of people around the globe and substantiates an enormous economic burden to society. Digital tools such as mobile Health (mHealth) applications have the potential to increase patient engagement, knowledge about the disease, and adherence to treatment recommendations. Digital health companions represent a new kind of digital tool aiming to support patients throughout their course of periodontal care. This paper presents the study protocol of the Paro-ComPas project which aims to co-develop and evaluate a digital patient companion application (“app”) to empower patients along their journey with periodontitis. As a first step, a qualitative study design encompassing semi-structured interviews with patients and experts as well as focus group discussions (FGD) will be used. Patients in different stages of periodontal care will be recruited from dental practices across Germany and are invited to share their experiences and opinions about their care and potential areas for support. Experts from relevant areas (e.g., mHealth, behavior change psychology, oral health, and dental hygiene) will be interviewed to map a holistic view on the current delivery of care and best practices of mHealth development. After setting up a minimal viable product (MVP) based on a requirements analysis, FGDs with patients will take place to incorporate user feedback and finalize the development of the prototypic app. The prototypic app will then be evaluated in a randomized, multi-center clinical trial in comparison with the current standard of care. Finally, a comprehensive implementation roadmap will be developed together with all relevant stakeholders. This comprehensive approach will allow us to map the patient journey and develop a digital health companion tailored to the needs of patients with periodontitis using an already existing indication independent medical companion toolbox. Novel insights into patients’ knowledge and perception of periodontal disease as well as barriers in adherence to periodontal care pathways will be provided. This knowledge will be converted in a systematically tailored companion app to serve the needs and preferences of people to better address periodontitis. The results from the clinical trial will provide unique insights into the extent to which the patient companion app contributes to adherence to periodontal care. Although mHealth applications have become popular in recent years, only few apps focusing on promotion of oral health have been released so far. Our study presents a novel and comprehensive approach to both co-developing and evaluating a proof of concept for a digital health companion for patients with periodontitis.

## Introduction

Worldwide, 11% of the world's population or 743 million people suffer from periodontitis (1). Periodontitis is a chronic inflammatory disease resulting in the destruction of tooth-supporting tissues and tooth loss. The disease is one of the main reasons for loss of teeth in adults worldwide (2) and can have a negative influence on chewing, oral aesthetic and quality of life (QoL). Periodontitis substantiates considerable treatment costs and contributes to exacerbation of social inequalities in oral health (1). Periodontal disease is also associated with and shares common risk factors with systemic diseases which lead to premature death, such as type 2 diabetes and cardiovascular diseases (3). Interdependencies between periodontitis and diabetes complicate the treatment of both diseases and increase the treatment costs (4). On a global scale, periodontitis heavily contributes to the cost of 544 billion USD that are attributed to dental diseases yearly (5). For Germany, loss in productivity due to periodontitis is estimated at 3.2 billion Euro yearly, not including cost of tooth loss (5). However, despite its high disease burden, many people with periodontitis do not seek comprehensive periodontal care (1, 6).

In 2020, the European Federation of Periodontology (EFP) published a clinical guideline for the treatment of stage I-III periodontitis (7). Within this guideline, treatment is approached based on the assessed stage of the disease, with each stage requiring different interventions. Instructing patients and motivating them to implement behavioral changes is described to be essential throughout the treatment course. To maintain periodontal stability, active therapy is followed by supportive periodontal care (SPC). SPC includes both preventive and therapeutic measures and takes place in regular and individually adapted intervals. Important components of SPC are monitoring of periodontal health, reinforcement of individual oral hygiene, and motivating patients to maintain risk factor control (7). The success of periodontal care thus significantly depends on engaging and empowering patients throughout the course of periodontal care. However, there is a risk that patients cannot comprehend the complex care path, endangering patient adherence over the course of treatment. Thus, the new EFP S3 level clinical practice guideline emphasizes the importance of communication skills and engaging communication. Such communication was identified as indispensable for behavior change in patients and the success of the new guideline. Yet, implementation gaps remain. It is necessary to understand behavior change from the patients' perspective and to emphasize the development of concretely actionable strategies for behavior change.

The reasons for low uptake and adherence to periodontal therapy are not well known. A discrete-choice experiment by the German Institute for Quality and Efficiency in Health Care revealed that patients have a strong preference concerning the prevention of tooth loss and the appearance of “long” teeth due to gingival recession (8). However, there is reason to assume a strong asymmetry between the objective need for care and the patients' perception of urgency for periodontal care. In part, this can be contributed to the course of periodontitis, which is a mostly slow and painless disease and often described as a “silent” disease. In later stages of periodontitis, a significant destruction of the periodontal ligament and the bone can be observed, leading to notable loosening of the teeth and tooth loss. In a study population showing a clinical prevalence of 75% of moderate to severe periodontitis, self-awareness of periodontal status was compared with clinical findings (9). The results indicate that in this study population 86% of participants subjectively did not consider having periodontitis. An earlier telephone survey among German citizens showed that there are deficits in periodontitis-related knowledge, including the definition of periodontitis, risk factors associated with the disease and effective preventive measures (10). A lack of awareness of one's own disease can lead to delayed start of therapy, e.g., when massive destruction of tissue has already occurred. Lacking awareness for the significance of the disease can also negatively affect therapy itself (11, 12). Comprehensive consideration of the affected patients' experiences, needs, expectations, and preferences is necessary to recognize the complex reasons for the insufficient use of care. Furthermore, there is a need to understand the reasons for low motivation and adherence to oral hygiene instructions and therapeutic measures. Only through a better involvement of patients in the different steps of therapy, awareness for the disease and use of health care services can be improved. This would also lead to an increase in the necessary motivation for better adherence and behavior change.

Digital applications present a promising approach to organize health care in a more participatory way and to address implementation gaps. In addition, patient empowerment, sharing of knowledge, and adherence can also be improved. Research suggests that patients who use digital tools such as electronic health records show an improved adherence to therapy, shorter hospital stays, increased survival, and an overall higher quality of life (13). Digital health companions present a new type of digital application. Tailored to the medical indication, they can support patients with helpful tools throughout the course of the therapy. One example is the application “mySugr” (14). Digital health companions can contain elements such as questionnaires, documentation/health records, reminders, and motivation. Therefore, a digital companion could also support patients with periodontitis by offering tailored support.

The project presented in this protocol, “Paro-ComPas”, investigates existing barriers contributing to low use of health care services, low motivation, and adherence. This investigation is based on a patient journey approach. Based on these findings, concrete approaches for a better involvement of patients in periodontitis care will be developed. These aim to increase the patients' awareness for the disease and help to shape care delivery in a more participatory manner. Over the course of the project, a proof of concept for a new digital health companion will be developed. Additionally, the application will be evaluated in order to study whether it is able to counteract the identified barriers and improve treatment outcomes by increasing patient engagement in periodontal care.

## Materials and analysis

**Figure 1:**
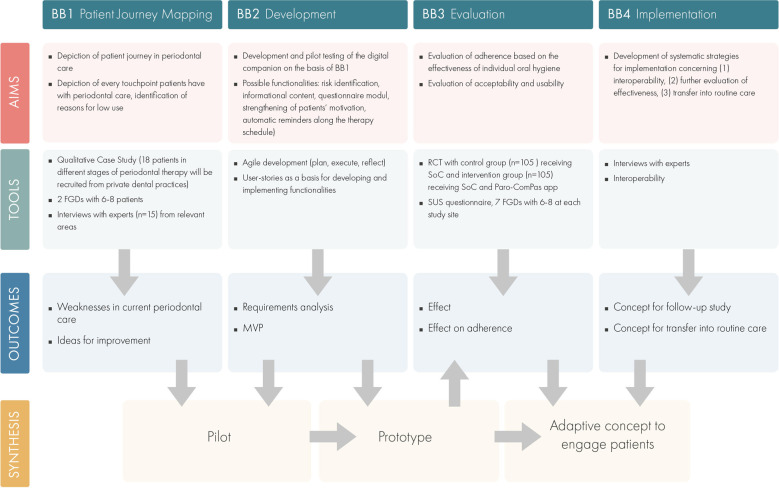
Overview of the building blocks and the study design.

The Paro-ComPas project is divided into four building blocks, which will be worked on consecutively. Figure 1 presents an overview of the building blocks and the study design.

### Building block 1: patient journey mapping

The first building block, “patient journey mapping”, aims to create an understanding for the journey a patient in periodontal care goes through. A special focus is put on possible barriers for optimal care and areas for support.

• In order to collect these insights, a qualitative approach will be used. First, patients in different stages of periodontal therapy will be recruited from six dental practices across Germany. Possible study participants will be identified through an online screening survey. The survey includes questions on the patients' sociodemographic data, health-related behavior (e.g., smoking), and oral health quality of life (OHIP-G5) (15). The completed surveys will be analyzed descriptively, and potential study participants will be identified using a maximum variance/heterogeneity sampling strategy based on their responses to the questions in the screening survey (16). Based on their preferences, potential study participants will be invited to participate in either (1) one-time semi-structured interviews or (2) focus group discussions (FGD). The semi-structured interviews will be conducted by a researcher with experience in qualitative interviewing and will be carried out telephonically. The planned sample size for the semi-structured interviews is *n* = 18. The interview guide will ask participants to describe their experiences with periodontal care, barriers they have encountered, and where they see a need for additional support from a digital companion. Two FGDs are planned with six to eight patients each (17). One FGD is set in advance of app development to discuss potential app functions in an open and interactive format. Results from this FDG will also be triangulated with the semi-structured interviews. Another FGD is set to take place as soon as a minimal viable product (MVP) has been developed. This allows for the collection of early feedback on the design and functions of the app.
• In addition to interviews and FGDs with patients, experts from relevant fields (e.g., oral health, dental hygiene, behavior change psychology, mHealth) will also be interviewed. Experts will be recruited using a purposive sampling strategy and will be contacted *via* email. Interviews will be conducted telephonically or *via* video chat. Based on the interviewee's area of expertise, the interview guide will include questions on helpful functions in the app or on best practices in mHealth development and behavior change psychology.
• All qualitative data will be audiotaped, anonymized, and transcribed. Transcripts will be analyzed using thematic analysis (18).

### Building block 2: companion development process

The aim of this building block is to efficiently collect and define software requirements for the digital companion supporting patients in periodontal care and to develop a prototype using the already existing phellow seven companion toolbox (CTX) which is a quality assured indication independent framework to build interoperable, patient centric medical companion apps (19). The overall approach in this building block is based on an iterative approach to consider new requirements quickly.

The CTX framework comes with an interoperability layer providing connectivity towards information systems like electronic health records (EHR) and to wearables by common international healthcare standards and profiles like HL7 FHIR and IHE. CTX is designed to build patient-centric medical companions. Therefore, it provides basic functionality which can be included such as electronic medical records (EMR), questionnaires, diaries, appointments, and patient journey mapping. It is modular, extensible and quality assured and can be adapted to certain layouts and user experience designs.

Following the agile principles as described in Agile Manifesto (20, 21) allows to use multiple agile methods as Scrum and Lean, repeat software development activities in response to changes and frequently gathering user feedback. The first phase of this process is the discovery phase including understanding and defining the problems and needs of stakeholders from different perspectives (patients, physicians/domain experts) using Design Thinking Methods and creative requirements workshops (22). Usually, this phase takes into account the insights from the patient journey mapping (1.1) and results in agile concepts like User Stories or Personas (23). User Stories are part of requirements planning and key for incremental development and the following development phases (prototyping, testing).

### Building block 3: evaluation

The aim of the third building block (“evaluation”) is to evaluate the effectiveness of the digital companion measured by increased adherence to oral hygiene recommendations. Another aspect to be evaluated in this building block are usability and acceptability of the app.

#### Evaluating the effect on adherence to oral hygiene

One goal of the Paro-ComPas app is to improve patients' adherence to oral hygiene recommendations. To evaluate if this goal can be met by using the digital companion, a multicenter, controlled, two-armed, randomized trial (RCT) will be conducted. The intervention arm will use the MVP of the Paro-ComPas app in combination with the standard of care (SoC) for periodontitis. Results will be compared to a control group receiving SoC. The trial's primary endpoint is defined as the Gingival Bleeding Index (GBI). Secondary endpoints are: (1) share of patients with ≤4 locations with pocket probing depths of ≥5 millimeters after systematic periodontitis therapy, (2) oral health-related quality of life (measured with OHIP-G14 (15)), and (3) awareness of periodontitis (questionnaire instrument currently in development by the study team). The necessary sample size to show a potential statistical effect was calculated at 210 participants (assumptions for sample size calculation: effect size = 20% (24), alpha = 0.05, power = 80%, drop-out rate = 20%).

Participants will be recruited from seven academic dental hospitals in Germany. At each study site, half of the participants will be randomized to the control (*n* = 105) or the intervention group (*n* = 105). Participants in the intervention group will receive a link to download the Paro-ComPas app and training materials. The overall length of the RCT per participant is set at 1 year and accompanies the participant's treatment course through active treatment and SPC. GBI and probing depths will be evaluated in a standardized manner at three time points: (1) start of active treatment, (2) during re-evaluation (3–6 months after non-surgical periodontal treatment), (3) during SPC. At the time of these study visits, participants will be asked to fill in an online survey. The survey will consist of the OHIP-G14 and a questionnaire about awareness of periodontitis.

Descriptive as well as inductive and panel-regression models will be used for statistical analysis.

#### Evaluating acceptability and usability

To evaluate the usability of the newly developed digital companion, participants in the intervention group will be asked to fill in an additional online survey based on the System Usability Scale (SUS) (25). Furthermore, FGDs with 6–8 participants from the intervention group will be conducted at each study site. FGDs will be conducted with a heterogenous sample, consisting of (1) participants showing adherence, (2) non-adherent participants, and (3) adherent participants, who dropped out of the trial. FGDs will be based on a semi-structured interview guide. The interview guide will include questions on acceptability, based on the Universal Theory of Acceptance and Use of Technology (UTAUT) (26) framework. Other questions will discuss barriers to using the Paro-ComPas app.

### Building block 4: implementation strategy

This last building block aims to develop a sustainable strategy for the implementation of the Paro-ComPas app. The development of the implementation strategy is guided by three aspects.

#### Interoperability

Given the chosen methodology for software development, concrete functionalities of the companion will be developed over the course of the project and are not pre-defined. Still, interoperability with other applications and products was defined as a possible technical goal for this project in advance. Possibilities to increase patient engagement in oral health care (e.g., improved transfer of knowledge, stronger motivation) that are developed and evaluated within this project, could be able to be linked to other concepts and products in a modular manner. Through interface compatibility with other technical devices, such as artificial intelligence (AI)-supported “smart” toothbrushes, the functionalities of the Paro-ComPas app could be enhanced to further increase patients' adherence to oral health recommendations. One way to successfully implement the Paro-ComPas app could be to build in broad interoperability with other mHealth applications. Therefore, Paro-ComPas should offer the necessary interfaces and follow common standards for data exchange and integration.

#### Further evaluation of the effectiveness

The digital companion Paro-ComPas will be developed to reach *technology readiness level 3* (27). Due to the time limitations of the funded project, it is not possible to evaluate the product's effectiveness in private dental practices. Hence, the lasting value of the presented approach as well as the effectiveness of the digital companion on periodontitis care and patient adherence can only be examined in a follow-up study. Over the course of the project, a concept for this follow-up will be prepared and published.

#### Transfer of the application into the statutory health insurance catalogue

Within this fourth building block, the necessary economic, legal, and technological steps for the transfer of the results into routine care should be explored. Interviews with experts from five areas are planned to include all relevant stakeholders. These experts include representatives from (1) health insurance companies, (2) the National Association of Statutory Health Insurance Dentists, (3) health services research, (4) the National Agency for Digital Health (*gematik*) (28)), and (5) software developers in the field of mHealth applications.

## Discussion

The presented study protocol entails a number of unique and innovative contributions to the knowledge base. First, the complete depiction of the patient journey in periodontal care will deliver relevant insights into current barriers in periodontal therapy. Second, our proposed digital companion could be able to address some of these barriers. On an individual level, the use of a disease-specific digital health companion could improve patient adherence and disease awareness. This could lead to improved oral health, quality of life, and treatment satisfaction. Through improved support provided by the digital companion, patients could become empowered and take on a more active role in their periodontal care. These possible developments complement periodontal care and can have a positive impact on dental care costs. Third, the clinical evaluation of the prototypic app will provide highly unique and early insights into the effectiveness on a hitherto not comprehensively researched mechanism of promoting periodontal and oral health. Not least, an innovative medical informatics development approach will be used for the development of the app. Interoperability with other tools or devices (e.g., AI-supported toothbrushes) and infrastructures (e.g., German telematic infrastructure) is observed as a guiding principle. Over the course of the project, necessary steps to pave the way for an implementation of the digital companion into routine care will be identified systemically and prepared. The learnings from this project will be highly useful for future development of similar digital tools in the oral health field.

Although the application in this project will be specifically developed and tailored to periodontitis, our project can also serve as an exemplary approach for the development of digital health companions for other diseases with high societal relevance, especially because the underlaying technology is built to scale towards digital companionship in other medical domains.

## Data Availability

The original contributions presented in the study are included in the article/Supplementary Material, further inquiries can be directed to the corresponding author/s.
